# Astroglial connexins and cognition: memory formation or deterioration?

**DOI:** 10.1042/BSR20193510

**Published:** 2020-01-10

**Authors:** Jin-Ting He, Xiao-Yan LI, Le Yang, Xin Zhao

**Affiliations:** 1 Department of Neurology, China-Japan Union Hospital, Jilin University, Changchun, 130033, Jilin Province, China; 2 Department of Endocrinology, The People’s Hospital of Jilin Province, Changchun 130031, China; 3 Department of Paediatrics, The First Hospital of Jilin University, Changchun, Jilin 130021, China

**Keywords:** Alzheimer disease, ATP, Connexin, gap junction, glutamate

## Abstract

Connexins are the membrane proteins that form high-conductance plasma membrane channels and are the important constituents of gap junctions and hemichannels. Among different types of connexins, connexin 43 is the most widely expressed and studied gap junction proteins in astrocytes. Due to the key involvement of astrocytes in memory impairment and abundant expression of connexins in astrocytes, astroglial connexins have been projected as key therapeutic targets for Alzheimer’s disease. On the other hand, the role of connexin gap junctions and hemichannels in memory formation and consolidation has also been reported. Moreover, deletion of these proteins and loss of gap junction communication result in loss of short-term spatial memory. Accordingly, both memory formation and memory deteriorating functions of astrocytes-located connexins have been documented. Physiologically expressed connexins may be involved in the memory formation, while pathologically increased expression of connexins with consequent excessive activation of astrocytes may induce neuronal injury and cognitive decline. The present review describes the memory formation as well as memory deteriorating functions of astroglial connexins in memory disorders of different etiology with possible mechanisms.

## Introduction

In the central nervous system, the communication among different cells and extracellular space is mediated through gap junction and hemichannels. Gap junction channels help in intercellular communication between different cells, while the interaction between cells and extracellular space is mediated through hemichannels [1,2]. The term hemichannels signify that gap junction is composed of two hemichannels. The gap junctions and hemichannels allow the direct transfer of small signaling molecules, second messengers, metabolites, ions, and electrical signals from cell to cell, and cell to extracellular space [3]. Interestingly, both gap junctions and hemichannels are mainly composed of connexins, which are integral membrane proteins forming high-conductance plasma membrane channels. Among 20–21 different types of connexins, connexin 43 is one of the major gap junction proteins in astrocytes [4].

Dementia refers to a deterioration in memory, thinking, behavior that impairs the ability to perform daily functions. Dementia is more common in elderly persons; however, it is not attributed to normal aging process. There are many types of dementia and Alzheimer’s disease is the most common form of dementia (60–70%). The other forms of dementia include vascular dementia, dementia with Lewy bodies and frontotemporal dementia. The latter is an umbrella clinical term that includes a group of diseases characterized by neurodegeneration in the cortical and temporal regions. Symptomatically, it is characterized by dementia along with behavior and language deficits and it is more common patients younger than 65 years [5,6]. Studies have shown the key role of non-neural cells, mainly astrocytes in the pathogenesis of neurodegenerative diseases including Alzheimer’s disease [7]. Indeed, astrocytes are found to be the key mediators of cognitive impairment [8]. The changes in astrocyte gap junction communication in response to β-amyloid have been documented [9]. Considering the key role of astrocytes in memory impairment and abundant expression of connexins in astrocytes, astroglial connexins have been projected as key therapeutic targets for Alzheimer’s disease, dementia with Lewy bodies and HIV-induced dementia [10–12]. However, there have not been studies exploring the role of connexins and gap junction in the pathogenesis of frontotemporal dementia.

In contrast with the studies showing the deleterious effects of connexins on learning and memory, the role of connexin gap junctions and hemichannels in memory formation and consolidation has also been reported [13]. It has been shown that the deletion of these proteins and loss of gap junction communication results in a loss of short-term spatial memory [14,15]. Accordingly, both memory formation and memory deteriorating functions of astrocytes-located connexins have been documented. The present review describes the memory formation as well as memory deteriorating functions of astroglial connexins in memory disorders of different etiology with possible mechanisms.

## The key role of connexins in memory formation

There have been a number of studies documenting that connexins-associated gap junction and hemichannels are essential for normal learning and memory [13–16] (Table 1). Even the involvement of gap junction networks in the mushroom bodies in visual learning and memory in Drosophila has been reported [17,18]. Indeed, the gap junction communication is critical in inducing long-term potentiation (LTP) by stimulating the excitatory connections between piriform cortex pyramidal neurons. The blockade of connexin 43 hemichannels has been associated with a significant decrease in LTP and impairment in memory consolidation [13]. The key role of connexin 43 in learning and memory has been described in a number of studies. Interference with connexin 43 hemichannels has been shown to influence the hippocampal spatial memory. Microinfusion of transactivator of transcription-linked Gap19 (TAT-Gap19), which specifically inhibits hemichannels, into the brain ventricle significantly impaired the spatial short-term memory in a delayed spontaneous alternation Y maze task. Accordingly, it was suggested that the presence of connexin 43 channels is required for spatial short-term memory [14]. Moreover, the role of these gap junctions is also delineated in fear learning and the blockade of neuronal gap junctions within the dorsal hippocampus has been associated with impairment in context-dependent fear learning and memory [15]. The role of astroglial connexin 43 hemichannels in fear memory consolidation has also been defined. Microinfusion of TAT-Cx43L2 and Gap27, inhibitors of connexin 43 hemichannels, into basolateral amygdala led to the development of amnesia during fear conditioning suggesting the role of these channels in memory consolidation [16].

**Table 1:** Summarized studies showing the key role of connexins in memory formation

S. No	Study plan	Key findings	References
1.	Inhibition of brain hemichannels by transactivator of transcription-linked Gap19 (TAT-Gap19)	Impairment in the spatial memory in Y maze task	[14]
2.	Microinfusion of TAT-Cx43L2 and Gap27, inhibitors of connexin 43 hemichannels, into the basolateral amygdala	Development of amnesia during fear conditioning	[16]
3.	d-Galactose-induced AD in ovariectomy-subjected mice	Disruptions in the structural integrity of neuronal-glial units and decreased expressions of connexin 43	[19]
4.	Depletion of drebrin, a binding partner of the connexin 43, using siRNA	Cognitive decline along with a decrease in connexin 43	[20–23]
5.	Comparison of slices of the hippocampal CA1 area obtained from connexin 36 knockout and normal mice	Significant reduction in LTP in the hippocampal slices obtained from connexin 36 knockout mice	[26]
6.	Evaluation of memory in connexin 36 knockout mice	Impairment in spatial short-term memory in these mice	[27]
7.	Evaluation of changes in connexin levels during memory consolidation in the passive avoidance test	Up-regulation of connexins 36 and 45 mRNAs in the hippocampus	[28]
8.	Chronic cerebral hypoperfusion by permanent occlusion of carotid arteries-induced vascular dementia	The decrease in the mRNA and protein expression of connexins 32 and 36 in the hippocampus	[30]

It has been shown that there is a disruption of neuronal-glial-vascular units in the hippocampus region in a mouse model of Alzheimer’s disease. In ovariectomy-subjected mice, injection of d-galactose for eight weeks (to induce Alzheimer’s disease) led to reactive astrogliosis (increase in glial fibrillary acidic protein reactivity) and destroyed astrocytic domain organization in the hippocampus. The biochemical analysis revealed that these mice had decreased expressions of connexin 43 suggesting that disruptions in the structural integrity of the neuronal-glial units may contribute to neurodegeneration [19]. The association of connexin 43 in Alzheimer’s disease may be possibly linked through drebrin, an actin-binding protein. Drebrin is a binding partner of the connexin 43 that helps in linking the gap junctions to the sub-membrane cytoskeleton and hence, it helps in maintaining connexin 43 containing gap junctions in their functional state. The depletion of drebrin in cells using siRNA has been associated with impairment in gap junction communication and degradation of connexin 43 [20]. There have been several studies suggesting that the decrease in the levels of drebrin in Alzheimer’s disease [21,22]. Accordingly, it may be possible that the decrease in the levels drebrin may impair connexin 43 associated gap junction communications that may contribute to cognitive decline in Alzheimer’s disease [23]. The maintenance of connexin 43 expressions in astrocytes also helps in preventing neuronal injury in response to oxidative stress. It is well reported that the major antioxidant in the central nervous system, i.e. reduced form of glutathione (GSH) is primarily synthesized and released by astrocytes [24]. Moreover, monomeric form of β-amyloid is reported to stimulate more effective release of GSH from the cultured cortical astrocytes than oligomeric Aβ or fibrillary Aβ. Moreover, it is also shown that β-amyloid facilitates connexin 43 hemichannel opening in the astrocytes to release GSH. Accordingly, it may be hypothesized that less aggregated form of β-amyloid may increase the release of GSH from astrocytes to provide protection from the oxidative stress in the early stage of Alzheimer’s disease [25].

Apart from the key role of connexin 43, studies have also shown the role of other connexins including connexin 36, 32, 31.1 and 45 in learning and memory [26–29]. The deletion of gap junction protein, connexin 36, has been associated with defective learning and memory. Indeed, it is shown that there is a significant reduction in LTP in the acute slices of the hippocampal CA1 area, obtained from connexin 36 knockout mice in comparison with wild-type mice suggesting that learning and memory deficiencies in connexin 36 knockout mice are due to reduction in LTP [26]. Another study has shown the impairment in spatial short-term memory in connexin 36 knockout mice, thereby suggesting that connexin-36 associated gap junctions are required for normal spatial coding in the hippocampus and short-term spatial memory [27]. Another study has shown the key role of connexin 36 in memory consolidation by showing a rapid up-regulation of connexins 36 and 45 mRNAs in the hippocampus (within 30 min) in a passive avoidance paradigm in rats [28]. In response to chronic cerebral hypoperfusion (permanent occlusion of carotid arteries)-induced injury, a significant decrease in the mRNA and protein expression of connexins 32 and 36 in the hippocampus region of rats has been reported. Moreover, the density of gap junctions on the cell membranes was found to be significantly lower along with an increase in the spaces between the gap junctions. Importantly, these changes in connexin expression and gap junctions were correlated to the defects in spatial learning and memory ability [30]. Moreover, a research study has shown that knockout of connexin 31.1 in mice leads to impairment in object learning suggesting the role of these connexins in memory formation [29].

At present, studies are not available describing the direct linkage of mutations in connexin 43 and the development of dementia in humans. Accordingly, there is a need for future studies to explore the linkage of single-nucleotide polymorphism (SNP) or mutations in connexin 43 with dementia may be identified in pedigree or Genome-wide association studies (GWAS).

## Increase in connexins produces deleterious effects and its knockout or blockade improves memory

In contrast with above-described studies showing the key role of connexins in memory formation, there have been a large number of studies showing that there is an overexpression of connexins in memory disorders of different etiology and the blockade of connexin-associated gap junction communication leads to the preservation of memory [31–33].

### Deleterious effects of connexins in Alzheimer’s disease

Studies have shown the key role of connexins-associated gap junctions and hemichannels in β-amyloid-mediated neuronal cell death in Alzheimer’s disease [34,35]. The levels of connexin 43 may be increased in the brain either due to an increase in transcription or decrease in degradation. One of the pathways involved in degradation of connexins involves activation of ubiquitin–proteasome pathway, which in turn may be modulated in the presence of exogenous substances or internal biochemical alterations. However, increase in the connexin levels in memory deteriorating studies has been attributed to increase in its expression secondary to up-regulation of mRNA and protein synthesis (discussed below).

#### Expression of connexin 43 is increased in the astrocytes around β-amyloid plaques

There have been a number of studies showing an increase in the expression of connexin 43 in the patients as well as in the models of Alzheimer’s disease [36,37]. More importantly, this increase in connexin expression is particularly detected in most reactive astrocytes located at amyloid plaques [38]. In one of the earlier studies in this area, Nagy et al. demonstrated the relationship between connexin 43 and Alzheimer’s disease by studying the distribution pattern of connexin 43 and amyloid plaques in the brains of Alzheimer’s disease patients. Using light microscopy, it was shown that the cortical areas containing amyloid plaques exhibited an increase in immunostaining for connexin 43. Using electron microscopy, it was revealed that connexin 43 was localized to the astrocytic gap junctions in these brains [39]. Later, studies in animal models of Alzheimer’s disease also revealed the higher expression of connexin 43 in the brain. In transgenic mice with overexpressing β-amyloid precursor protein (APP) and presenilin1 (PS1) (models of Alzheimer’s disease), an increase in astroglial connexin immunoreactivity specifically around the β-amyloid plaques was documented. Indeed, the changes in the immunostaining pattern of connexin 43 around the β-amyloid plaques started to appear in 4-month-old mice and an overall increase in connexin expression was detected in 18-month-old APP/PS1 mice [40]. Other studies also found the higher expression of astroglial connexin 43 around the amyloid plaques in APP/PS1 mice [41].

In another Alzheimer’s mouse model (lacking inducible nitric oxide synthase), an increase in the phosphorylation form of connexin 43 was reported in the astrocytes at the age of 42 weeks [42]. In comorbid rat models of Alzheimer’s disease (Aβ-induced toxicity) and stroke, the pathological changes including hippocampal atrophy, cortical degeneration and cognitive deficits have been associated with the bilateral induction of connexin 43 along with reduced neuronal survival [43,44]. In APPPS1 mice (model of Alzheimer’s disease), an increase in astrocytic hyperactivity and reactive astrogliosis was most pronounced around the β-amyloid plaques [45]. In a murine model of familial Alzheimer’s disease i.e. APPswe/PS1dE9 mice, the activation of astrocytic hemichannels (connexin 43 as the main contributor) in acute hippocampal slices containing Aβ plaques has been reported [46]. In a recent study based on the transcriptomic and proteomic datasets from post-mortem AD and normal control brains revealed that GJA1 is strongly associated with β-amyloid deposition and cognitive dysfunctions [47].

#### Pharmacological inhibition/knockout of connexin 43 ameliorates β-amyloid associated cognitive impairment

Considering the role of gap junctions and hemichannels in the pathogenesis of Alzheimer’s disease, scientists have attempted to explore the therapeutic potential of pharmacological inhibitors of gap junctions. Treatment with cannabinoids has been shown to reduce the activation of astrocytic connexin 43 hemichannels. Moreover, cannabinoids reduced the release of gliotransmitters (excitotoxic glutamate and ATP) from astrocytes and prevented β-amyloid-induced neuronal damage in the hippocampal slices [32]. Ren et al. crossbred GFAP (glial fibrillary acidic protein)-connexin 43 knockout mice with APP/PS1 mice to obtain APP/PS1/GFAP-Cx43 knockout mice. In other words, they developed the model of Alzheimer’s disease with the specific knock out of connexin 43 and thereafter, they reported that the cognitive function of 12-month-old mice with specific deletion of astroglial Cx43 was significantly preserved. It was also revealed that in these mice there was a decrease in the GFAP expression, increase in the number of synapses without any significant alteration in β-amyloid plaques deposition. It suggests that the deletion of astroglial connexin 43 in APP/PS1 mice may help in preserving cognitive functions by inhibiting astrogliosis, up-regulating synaptic function without affecting the formation of amyloid plaques. Interestingly, re-expression of connexin 43 in these mice led to cognitive decline marked with astrogliosis and decrease in the number of synaptic functions, which suggests the key role of astroglial connexins in the neuronal dysfunctions and cognitive decline [41]. Other studies have shown that the astroglial targeted genetic knocking-out of connexin 43 in APPswe/PS1dE9 diminishes the release of gliotransmitters (such as ATP and glutamate) and abolish β-amyloid associated damage to the hippocampal neurons [46]. In contrast with the above-described finding that knockout of connexin 43 does not inhibit β-amyloid deposition [46], the *in vitro* study has shown the anti-amyloidogenic actions of a gap junctional blocker, carbenoxolone [48]. It may be possible that *in vitro* anti-amyloidogenic actions of a gap junctional blocker may not be translated into *in vivo* effects of decrease in β-amyloid deposition. Nevertheless, treatment with carbenoxolone has been shown to attenuate *icv* injection of Aβ42 oligomers-induced decline in cognitive functions secondary to decrease in oxidative damage [33].

#### Possible mechanisms involved in connexins-mediated memory impairment

##### Purinergic pathway

**Figure 1:**
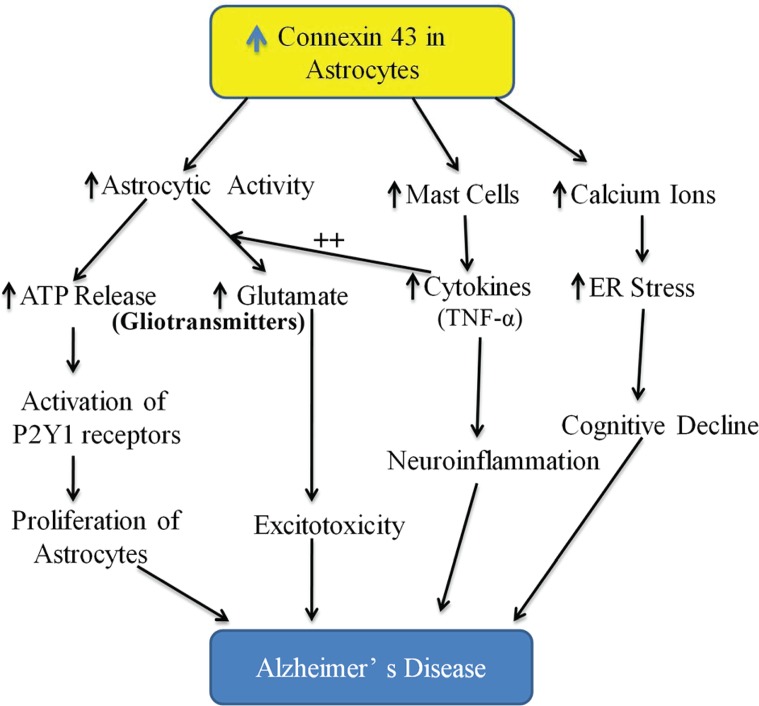
Proposed schematic representation of the role of astroglial connexin 43 in inducing memory deterioration in Alzheimer’s disease in association with other mediators An increase in the expression of connexin 43 on astrocytes may increase the activity of the astrocytes and in turn, astrocytes may respond by increasing the release of gliotransmitters including ATP and glutamate. ATP may activate P2Y1 receptors localized on astrocytes to further increase the proliferation of astrocytes in an autocrine manner. The release of glutamate, which induces excitotoxicity, may be potentiated in the presence of mast cells-derived cytokines such as TNF-α. Interestingly, the activation of mast cells may also be due to an increase in the connexin 43 expression on astrocytes. The induction of neuroinflammation in the presence of cytokines also contributes to the pathophysiology of dementia. An increase in intracellular calcium ions in response to an increase in connexin 43 expression may lead to the development of ER stress, which may be another mechanism contributing to the pathophysiology of Alzheimer’s disease.

Studies have shown the key role of ATP and other purine activated-purinergic receptors in Alzheimer’s disease [49,50]. It has been shown that overexpression of connexin 43 interacts with purinergic receptors, particularly, P2Y1 in inducing a cognitive decline in transgenic model of Alzheimer’s disease. Indeed, treatment with P2 (purinergic) receptor antagonist, pyridoxalphosphate-6-azophenyl-2′,4′-disulfonate, P2Y1 receptor blocker, MRS2179 and connexin channel inhibitor, carbenoxolone was shown to reduce the fraction of hyperactive astrocytes and improve cognitive function in APPPS1 mice [45]. It has been well documented that the gap junctions participate in inducing the ATP release from astrocytes [46,51–54]. Therefore, it may be possible that the increase in ATP release through connexin channels may lead to activation of P2Y1 receptors, which may be manifested in the form of astrocytic hyperactivity (Figure 1) and associated cognitive decline in APPPS1 mice [45]. There have been studies suggesting that down-regulation of P2Y1 receptors leads to transformation of astrocytes to a neuroprotective phenotype [55].

##### Mast cells

Mast cells are the important source of inflammatory mediators in the brain and their interactions with glial cells and neurons release the mediators including cytokines, proteases and reactive oxygen species [56]. Since there is an important role of neuroinflammation in the pathogenesis of Alzheimer’s disease, therefore, the role of mast cells in initiating neuroinflammation and inducing a cognitive decline in Alzheimer’s disease has been described [57]. Indeed, the distribution of mast cells near to the amyloid plaques has been described. Moreover, it is also described that the number of mast cells is increased in the hippocampal and cortical areas even before the deposition of amyloid plaque starts in APPswe/PS1dE9 mice. It possibly suggests that mast cells may act as early sensors of amyloid peptide and lead to recruitment of other inflammatory cells to initiate neuroinflammatory in Alzheimer’s disease. Regarding the interrelation between mast cells and connexins in cognitive decline, it was shown that connexin 43 hemichannels are required for Aβ25-35 to induce mast cell activation and histamine release in the brain slices of mice [58] (Figure 1).

##### Glutamate

Glutamate is an excitatory neurotransmitter and its excessive release has been found to induce neurodegeneration and cognitive impairment [59,60]. Within the brain, astrocytes play an important role in maintaining the glutamate homeostasis [61] and it has been shown that astrocytic connexin hemichannels control the release of glutamate from the astrocytes into the extracellular space [46,54]. Indeed, the release of glutamate via astroglial connexin 43 hemichannels is shown to mediate neuronal death [34,62]. The release of glutamate from astrocytes may further be potentiated in the presence of proinflammatory cytokines such as TNF-α as the latter has been shown to induce neurotoxicity by increasing the glutamate release from hemichannels of activated microglia in an autocrine manner [53,63]. Accordingly, it may be possible that increased expression of connexins in astrocytes during Alzheimer’s disease may lead to the excessive release of glutamate through hemichannels to induce neurodegeneration and cognitive decline (Figure 1).

##### Increase in calcium and endoplasmic reticulum stress

An increase in calcium levels and induction of endoplasmic reticulum (ER) stress are critical in the pathogenesis of Alzheimer’s disease [64,65]. It has been postulated that there is an interrelationship connexin 43, an increase in calcium levels and induction of ER stress. Indeed, in *icv* injection of streptozotocin-induced Alzheimer’s disease model, a correlation was described between increase in calcium levels, development of ER stress (increase in expression of ER stress markers like GRP78, GADD), activation of astrocytes and increase in the expression of connexin 43 [66,67]. There have been other studies showing the correlation in development of ER stress and increase in the expression of connexin 43 in different diseases [68]. Accordingly, it may be proposed that an elevation in calcium levels in the astrocytes may induce the development of ER stress and increase the expression of connexin 43 to alter cognitive function in Alzheimer’s disease (Figure 1).

### Deleterious effects of connexins in HIV-induced Dementia

The development of HIV-Associated Neurocognitive Disorders (HANDs) is one of the major complications of AIDS and cognitive impairment is found in approximately 50–60% of HIV-infected patients [69,70]. It has been shown that HIV-associated deleterious effects in the brain, particularly the neuronal loss is mediated through an increase in connexin 43 expression [71]. Unlike other viruses, HIV is shown to increase the expression of connexin 43 on the astrocytes, which is mainly attributed to the presence of HIV-tat protein. The latter binds to the connexin 43 promoter region to increase the mRNA and protein expression of connexin 43 along with gap junctional communication [72].

Regarding the deleterious effects of connexins in HIV-induced neurotoxicity, it has been demonstrated that the endogenous gap junctions and hemichannels of the host system help in amplifying HIV toxicity by spreading HIV to non-infected astrocytes, even in the absence of viral replication [73,74]. Using transgenic mice with specific deletion of connexin 43 in the astrocytes (hGFAP-cre Cx43^fl/fl^), the role of gap junctions in the propagation of HIV-infected astrocytes was demonstrated. It was shown that microinjection of few HIV-infected human astrocytoma cells (U87-CD4-CCR5) led to significant neuronal apoptosis in connexin 43 expressing mice, whereas hGFAP-cre Cx43^fl/fl^ mice (connexin 43 negative) were relatively resistant to the toxic effects of U87-CD4-CCR5 cells. It suggests the importance of the presence of connexin 43 on the astrocytes in HIV-induced neurotoxicity. This contention was further supported by the finding showing that the injection of gap junction blocker, 18α-glycyrrhetinic acid, and connexin 43 blocking peptide attenuated HIV-infected astrocytoma-induced neuronal apoptosis in connexin 43 positive mice [75]. Apart from it, it was also shown that HIV infection may also be propagated from the infected pericytes of the blood–brain barrier to other cells through gap junction/hemichannels-mediated intercellular communication. It has been demonstrated that HIV infection increased the expression of connexin 43 on the brain pericytes and inhibition of gap junctions by carbenoxolone was shown to prevent spreading of HIV infection within the brain [76].

Apart from spreading the HIV infection, connexins may also be involved in inducing neuronal injury in response to HIV infection. Indeed, in a mixed culture of neurons and astrocytes, HIV infection of astrocytes was shown to significantly reduce the number of neuronal processes. This neuronal loss was directly correlated to the opening of connexin43 hemichannels and an increase in the secretion of dickkopf-1 protein (DKK1) [77]. DKK1 is an endogenous, soluble inhibitor of Wnt signaling [78] and its expression has been found to be increased in transgenic mouse models of neurodegenerative disease including in Alzheimer’s disease [79]. Indeed, β-amyloid-induced synaptotoxicity is found to be DKK1 and Wnt-dependent. Therefore, it may be proposed that HIV infection of astrocytes induces dysregulation of DKK1 (increase in expression) by connexin 43-dependent mechanisms in contributing brain pathogenesis observed in HIV-infected individuals.

### Connexins participate in inducing dementia with Lewy bodies

Dementia with Lewy bodies is an age-related neurodegenerative disease with characteristic features of Alzheimer’s and Parkinson’s diseases. Lewy bodies are the intracellular protein aggregates containing α-synuclein and their excessive deposition in the hippocampal region is associated with progressive dementia in these patients [80]. There has been a study showing that α-synuclein modulates gap junctional intercellular communication and induces astrocyte dysfunction by directly binding to connexin 43 protein [81,82]. It has been shown that connexin-32 helps in up-taking α-synuclein oligomeric assemblies in neurons and oligodendrocytes. Using an *in vitro* model and transgenic mice, a significant correlation between an increase in expression of connexin 32 and accumulation of α-synuclein was reported. Furthermore, pharmacological and genetic strategies targeting connexin 32 led to reduction in α-synuclein uptake [83]. Considering the role of connexin 43 in the accumulation of α-synuclein and Lewy body deposition, it has been proposed that connexin 43 may serve as novel target to prevent dementia associated with Lewy bodies.

### Connexins in lipopolysaccharide-induced memory impairment

Intracerebroventricular injection of lipopolysaccharide induces neuroinflammation and leads to the development of cognitive impairment akin to Alzheimer’s disease. Accordingly, it has been employed as one of the chemical agents to induce cognitive impairment in experimental animals [84]. A research study identified that intracerebroventricular injection of lipopolysaccharide increases the gene expression of connexin 32 gap junction in the rat hippocampus [85] and it is possible that the resulting alteration in the gap junction communication in the hippocampus may contribute in cognitive decline. However, experimental studies are required to explore the direct relationship between lipopolysaccharide-induced cognitive decline and an increase in connexin 43 expressions.

### Role of connexins in Huntington’s disease

Along with the disturbance in the motor functions, there is a severe decline in cognitive functions in Huntington’s disease [86]. As in Alzheimer’s disease, scientists attempted to explore the relationship between Huntington’s disease and connexins by studying the expression of different connexins in the caudate nucleus and globus pallidus of the basal ganglia in the diseased brains. Among the different connexins, connexin 43 was the most abundantly expressed by astrocytes. Moreover, the density of connexin 43 was significantly higher in Huntington’s disease in comparison with normal brains. Along with it, the glial fibrillary acidic protein (GFAP) staining of astrocytes was also increased in the basal ganglia indicating a reactive astrocytosis around the degenerating neurons with an increased expression of astrocytic gap junctions (connexin 43) [87].

## Hypothesis

There have been contrasting studies regarding the role of connexins in memory formation [13–16] and memory deterioration [31–33]. Careful analysis of these studies helps in elucidating that most of the studies describing the memory formation role of connexins are done with an aim to unfold the basal role/function of these proteins in normal state. These studies describing the basal (physiological) function have described the essential role of connexin gap junctions and hemichannels in LTP formation, memory formation and consolidation. On the other hand, the studies describing the deleterious role of connexin gap junction and hemichannels are related to pathological conditions. In response to pathological stimulus including β-amyloid, increased expression of connexins in astrocytes may be associated with increased activation of astrocytes in the form of astrocytosis. Accordingly, excessive release of ATP from astrocytes with consequent activation of P2Y1 receptors, increase in glutamate release with an increase in intracellular calcium ions, oxidative stress and induction of endoplasmic reticulum stress may contribute in inducing neuronal damage and cognitive decline. Nevertheless, there has not been a single experimental study to explore the duality of connexins in memory, which can exactly explain the mechanisms responsible for dual role of connexins in memory. Accordingly, future studies should be directed to verify the hypothesis that the basal function of astrocytes-located connexins is to induce the memory formation, while increased expression of connexins with consequent excessive activation of astrocytes may induce neuronal injury and cognitive decline.

## Conclusion

Astrocytes-located connexins are involved in memory formation as well as in memory deterioration. Physiologically expressed connexins may be involved in the memory formation, while pathologically increased expression of connexins with consequent excessive activation of astrocytes may induce neuronal injury and cognitive decline.

## Data Availability Statement

The data will be available on request.

## Abbreviations

ER: endoplasmic reticulum
GWAS: genome-wide association study
LTP: long-term potentiation
SNP: single-nucleotide polymorphism
